# Potential Clinical Application of Determination of Bisphenols in Pericardial Fluid from Patients with Coronary Artery Disease

**DOI:** 10.3390/molecules31091442

**Published:** 2026-04-27

**Authors:** Tomasz Tuzimski, Kamil Baczewski, Viorica Railean, Daria Tarkowska, Małgorzata Szultka-Młyńska

**Affiliations:** 1 Department of Physical Chemistry, Faculty of Pharmacy, Medical University of Lublin, Chodźki 4a, 20-093 Lublin, Poland; 2 Department of Cardiac Surgery, Medical University of Lublin, Jaczewskiego 8, 20-093 Lublin, Poland; kamil.baczewski@umlub.edu.pl; 3 Department of Infectious, Invasive Diseases and Veterinary Administration, Institute of Veterinary Medicine, Nicolaus Copernicus University in Torun, Gagarina 7, 87-100 Torun, Poland; viorica.railean@umk.pl; 4 BioColl Team, Centre for Modern Interdisciplinary Technologies, Institute of Advanced Studies, Nicolaus Copernicus University, Wilenska 4, 87-100 Torun, Poland; dtarkowska@umk.pl (D.T.); mszultka@umk.pl (M.S.-M.); 5 Department of Environmental Chemistry and Bioanalytics, Faculty of Chemistry, Nicolaus Copernicus University, Gagarina 7, 87-100 Torun, Poland

**Keywords:** pericardial fluid, dispersive liquid–liquid microextraction (DLLME), LC–ESI–QqQ, bisphenol A and analogs, statistical analysis

## Abstract

Bisphenols may negatively impact human health, including the heart and circulatory system. It is crucial to determine the presence of these xenobiotics in biological samples, including pericardial fluid. Pericardial fluid was collected from patients with acute coronary syndromes (ACS), and with coronary artery disease during coronary artery bypass surgery. Bisphenol residues were identified and quantified in samples from 15 patients. Quantitative analysis of bisphenols in the samples was performed by LC–MS/MS on a triple quadrupole (QqQ) mass spectrometer and electrospray ionization (ESI−/ESI+) was applied in the negative and positive ion modes, respectively. The procedure was successfully applied to the biomonitoring of free forms of 14 bisphenols in pericardial fluid. After statistical examination of the relationships between the selected variables, it was concluded that while male subjects demonstrated higher Body Mass Index (BMI), longer procedural times, and earlier troponin release, female subjects exhibited later but more pronounced increases in CK and TrI, suggesting differences in kinetics and physiological response.

## 1. Introduction

According to the World Health Organization guidelines, cardiovascular diseases include disorders of the heart and blood vessels, such as coronary heart disease, cerebrovascular disease, rheumatic heart disease, and hypertension, among other conditions [1]. Out of the 18 million premature deaths (under the age of 70) due to noncommunicable diseases in 2021, at least 38% were caused by CVDs [1]. Unfortunately, the increase in cases of cardiovascular diseases continues. It is projected that by 2030, cardiovascular-related deaths are likely to increase up to 23.3 million [2]. More than 80% of health care dollars in the United States are now spent on treating chronic illness. 45% of cardiometabolic deaths (including heart disease, stroke, and diabetes mellitus) are now linked to poor diet [3].

A myocardial infarction is currently the most common cause of sudden hospitalisation and the leading cause of sudden death. A heart attack is a consequence and the final stage of a long-term coronary artery disease caused by atherosclerosis. The progression of atherosclerosis is determined by the presence of certain risk factors, such as lipid and carbohydrate metabolism disorders, hypertension, chronic inflammatory responses, smoking, a stress-inducing lifestyle, as well as other factors indirectly related to obesity and metabolic syndrome. Metabolic syndrome is the coexistence of metabolic disorders (abdominal obesity, arterial hypertension, dyslipidemia, insulin resistance, and high blood glucose level) that increase the risk of serious cardiovascular diseases and type 2 diabetes. In patients with advanced coronary atherosclerosis, disturbances in blood flow may occur, causing acute symptoms similar to those of a heart attack, but without the formation of necrotic foci in the ischaemic areas.

For this reason, contemporary medical practice approaches the diagnosis of coronary artery disease by considering its stages collectively, emphasising the “continuum of changes” that lead to acute ischaemia and myocardial necrosis. Describing myocardial infarction solely as an acute episode of ischaemia and myocardial necrosis does not fully encompass the challenges of diagnosis or clinical practice [4].

During a single contraction, the myocardium contracts in accordance with the “all-or-none” law. In an ischaemic and therefore hypoxic myocardium, metabolic, functional, and structural changes occur, while prolonged ischaemia leads to cell death. Hypoxia resulting from ischaemia inhibits energy-producing processes in mitochondria and leads to a reduction in intracellular ATP concentration [4].

A compensatory increase in glucose leads to increased concentrations of lactate and H^+^ ions, which results in acidosis. This, in turn, leads to the accumulation of Ca^2+^ ions, lactate, free fatty acids, acyl-CoA, and acylcarnitine within the cell.

Changes in cell membrane permeability can be observed as early as 15 min after the onset of ischaemia, leading to the release of certain cytosolic proteins into the interstitial space. During this earliest phase, increased concentrations of K^+^, Zn^2+^, Mg^2+^, and inorganic phosphates can be detected in coronary sinus blood, resulting from impaired function of membrane ion pumps [4].

After approximately one hour of ischaemia, the concentrations of adenosine, lactate, and pyruvate increase in coronary sinus blood due to anaerobic metabolism in cardiomyocytes.

After 1.5–2 h, increased concentrations of cardiomyocytes’ structural proteins in peripheral blood are observed. This is caused by the disintegration of cellular structure. The rate at which these changes develop depends on the initial condition of the myocardium [4].

The first phase, which usually lasts approximately three hours from the onset of ischaemia, is characterized by depletion of intracellular ATP stores and substrates required for its synthesis (ADP and AMP). This leads to the development of so-called myocardial stunning, which results in reduced cardiomyocyte contractility and impaired systolic function of the heart. These changes are reversible.

The second phase develops between the second and sixth hour of ischaemia. During this phase, glycogen reserves in cardiomyocytes are gradually depleted. As residual aerobic metabolism predominates, fatty acid oxidation predominates. However, fatty acid oxidation is a less efficient process and leads to depletion of limited oxygen reserves (this process consumes 10% more oxygen to produce the same amount of ATP compared to glucose metabolism) [4].

In addition to decreased ATP production, intracellular acidification as well as accumulation of Ca^2+^ occur. The increased Ca^2+^ accumulation activates a range of enzymes, including lipases, phospholipases, ATPases, and proteases. As a consequence, the function of the cell ion pumps is impaired, leading to the “leakage” of K^+^ ions and a rise in intracellular Ca^2+^ concentration [4].

Further consequences include damage to intracellular structures and advanced disintegration of the cell membrane, as well as structural changes such as an increasing number of small mitochondria and loss of myofilaments. As a result, the cardiomyocyte’s structure changes and begins to “look like” a fetal cell. Granules containing lipids, proteins, and calcium phosphates accumulate within the swollen cell due to the effects of fatty acids, acylcarnitine, and acyl-CoA on the cell membranes. This marks the beginning of irreversible changes. Only adenosine exhibits some protective effect [4].

Electrolyte imbalances cause neurophysiological and electrophysiological changes in the heart, as well as myocardial contractility disorders. A decline in diastolic function precedes the critical reduction in ATP concentration essential for cell viability.

Complete necrosis of all compromised cardiomyocytes occurs after 4 to 6 h (or later). Between 6 and 9 h after the onset of ischaemia, foci of myocardial necrosis develop, accompanied by the release of myocyte lysis products and infiltration of phagocytic cells. Proteins are released into the plasma and interstitial space. In the vicinity of the necrotic change, an inflammatory response develops, involving phagocyte infiltration of the damaged tissue. Tissue lysis occurs at the site of ischaemia, along with remodelling of the structure of the affected part of the myocardium. Complete reversal of ischaemia-induced changes is possible within 6 h of their onset [4].

In acute coronary syndrome, the fundamental condition for effective treatment is rapid diagnosis and initiation of appropriate therapeutic procedures aimed at restoring blood flow through the coronary vessels. Due to the rapid progression of changes in the ischaemic myocardium, inhibition of the processes leading to irreversible myocardial injury is of critical importance. Restoration of myocardial perfusion within six hours of the onset of ischaemia may result in reversal of hypoxia-induced changes [4].

The American College of Cardiology recommends the use of the terms acute coronary insufficiency or acute coronary syndrome (ACS) [4]. These terms describe a condition characterised by the occurrence of various episodes of myocardial ischaemia that may be transient or may lead to necrosis of fragments or areas of the myocardium, manifested by physical signs of ischaemia, characteristic changes on the electrocardiogram, functional disturbances, biochemical abnormalities, as well as changes visible on cardiac imaging tests.

Myocardial infarction is therefore one of the forms of ischaemic heart disease and very often results in the sudden death of a patient. However, patients who are appropriately treated and correctly diagnosed may avoid the occurrence of acute coronary syndrome and myocardial infarction.

Therefore, appropriate patient diagnostics and assessment of laboratory markers of risk factors for ischaemic heart disease are of crucial importance. These include metabolic markers, markers of hypoxia, necrosis, inflammation, coagulation disorders, renal dysfunction, and others (e.g., those observed in autoimmune diseases and polycythaemia) [5].

A new clinical guideline released by the *American Heart Association* includes new evidence and updated recommendations for managing patients with *acute coronary syndromes* (ACS). The guideline primarily focuses on the management of type 1 acute myocardial infarction—both *non-ST-elevation myocardial infarction* (NSTEMI) and *ST-segment elevation myocardial infarction* (STEMI)—and includes recommendations addressing initial evaluation and management of suspected acute coronary syndromes. Among the highlights are updated recommendations for pharmacologic care. Dual antiplatelet therapy with aspirin and an oral P2Y12 inhibitor is indicated for at least 12 months as the default strategy in patients with ACS who are not at high bleeding risk [5]. Additionally, high-intensity statin therapy is recommended for all patients with ACS. For those already on maximally tolerated statins and who have an LDL-C level ≥ 70 mg/dL (1.8 mmol/L), a nonstatin lipid-lowering agent such as ezetimibe, evolocumab, alirocumab, inclisiran or bempedoic acid is also recommended [5].

Laboratory diagnosis of myocardial infarction is based on the detection in the blood of the increase and decrease in specific proteins and enzymes released from the ischaemic area. The most important early markers of acute myocardial ischaemia include myoglobin, myosin light chains, troponin T, troponin I, tropomyosin, and the concentration of the cardiac fraction of creatine kinase (CK-MB mass assay).

Currently, specific monoclonal antibodies enable the direct measurement of the concentrations of these markers of myocardial necrosis. This allows us to determine the timing of the characteristic increase and subsequent decrease in marker concentrations following the onset of acute coronary syndrome. In myocardial infarction, the characteristic increase in the concentrations of individual markers—myoglobin, myosin light chains, troponin T, troponin I, tropomyosin, and CK-MB mass—occurs after specific time intervals: 1.5–2 h for myoglobin, 2 h for myosin light chains, 3–4 h for troponin T, 4–6 h for troponin I, 7–8 h for tropomyosin, and 4–6 h for the cardiac fraction of creatine kinase [4]. The decrease in the concentrations of these markers during myocardial infarction is also characteristic and occurs, following the onset of acute coronary syndrome, after the following time periods: 10 h for myoglobin, 10 days for myosin light chains, 9–10 days for troponin T, 7–8 days for troponin I, 10 days for tropomyosin, and 24 h for the cardiac fraction of creatine kinase [4].

During invasive treatment of coronary artery stenosis using percutaneous coronary intervention (PCI) or coronary artery bypass grafting (CABG), myocardial infarction may also occur, as indicated by an increase in troponin concentration.

The study by Fellahi et al. assessed the clinical relevance of using pericardial cTnI to evaluate perioperative myocardial damage in symptomatic patients undergoing elective CABG surgery. The study included 102 subjects with symptomatic coronary disease [6]. A similar study was conducted by Cihan et al. to evaluate the diagnostic potential of pericardial cTnI for detecting postoperative MI [7]. The study included 64 subjects undergoing elective CABG surgery. Tambara et al. described a study that measured troponin T (TnT) concentrations in serum and PF in 34 patients undergoing CABG for either unstable angina or stable coronary artery disease [8]. In another study, Kramer et al. investigated whether cardiac surgery resulted in the release of pro-oxidant and pro-inflammatory molecules into the pericardial fluid, and found that cTnI levels were significantly higher in pericardial fluid compared to serum [9]. Additionally, elevated pericardial fluid cTnI levels were associated with increased markers of oxidative stress [9]. In a study published by Butts et al. [10], inflammation in the pericardial fluid following cardiac surgery was examined in 19 patients who underwent CABG, a combined CABG and valve procedure, or a valve procedure alone.

Exposure to the emerging contaminant bisphenol A (BPA) is ubiquitous and associated with cardiovascular disorders. BPA’s effect as an endocrine disruptor is widely known but other mechanisms underlying heart disease, such as epigenetic modifications, remain unclear.

As described in the previous publication [11], few studies have been published on the harmful effects of bisphenols on the heart and circulatory system.

In their study, Lombo et al. observed that BPA exposure during early stages of development seriously affects heart development [12]. BPA has two mechanisms of action underlying cardiogenesis impairment: estrogenic and epigenetic, which have been identified by the overexpression of esr2b and the increase in histone acetylation (specifically in H3K9 and H4K12), respectively. The authors concluded that both mechanisms, which are closely related, might act in synergy and could be responsible for the upregulation observed in the transcription factor hand2, which is crucial for cardiac formation [12].

This is a continuation of our research published in early 2025 [11]. This study is the second to examine bisphenol residues in pericardial fluid collected from Polish patients with coronary artery disease undergoing coronary artery bypass surgery. As in the previous study, the manuscript also aims to link the frequency of bisphenols and their concentrations in pericardial fluid collected during coronary artery bypass surgery, and to explore the correlations between bisphenol frequency and concentrations and some common causes of cardiovascular (CV) system disease. Despite very invasive sampling, the data obtained during analysis may improve current knowledge about the toxicity of these analytes on the human cardiovascular system (e.g., in the pericardial fluid collected from patients diagnosed with aortic stenosis).

## 2. Results

**Figure 1:**
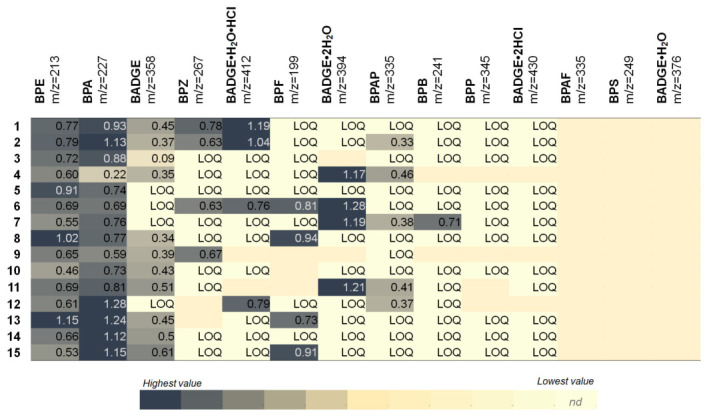
Distribution of bisphenol concentrations (ng/mL) in pericardial fluid samples from 15 patients diagnosed with coronary artery disease. Samples are labeled numerically from 1 to 15, corresponding to the individual specimens analyzed using LC–ESI–QqQ.

As demonstrated in Figure 1, a heatmap illustrates the concentrations of different bisphenol compounds that were detected in 15 samples.

BPE (*m*/*z* = 213) and BPA (*m*/*z* = 227) are consistently detected in all samples, typically at higher concentrations compared to the other compounds. For example, the maximum value of BPE is 1.15 ng/mL in sample 13, while the maximum value of BPA is 1.28 ng/mL in sample 12. BADGE (*m*/*z* = 358) has been detected in several samples at lower concentrations, which have in some cases fallen below the LOQ. BPZ (*m*/*z* = 267) and BPF (*m*/*z* = 199) are detected sporadically, with a few notable higher values. Indeed, the results indicate that sample 6, in which BADGE·2H_2_O reaches 1.28 ng/mL, exhibits one of the highest concentrations observed across all the samples analyzed. In contrast, BPAP, BPB, and BPP are detected less frequently, with most values being found to be below the LOQ. In the context of the present study, it is noteworthy that derivatives of BADGE (e.g., BADGE·H_2_O·HCl, BADGE·2H_2_O) and other compounds (e.g., BPAF, BPS, BADGE·H_2_O) are not frequently detected. Therefore, the variability of the sample is hereby demonstrated. It is evident that certain samples (5, 6, 13, and 15) contain high concentrations of compounds. Nevertheless, the composition of each sample is such that it contains either four or five bisphenols. BPE and BPA were identified as the most prevalent and abundant compounds among all samples, suggesting that they are either major contaminants or commonly released from materials associated with the samples. The majority of other compounds are present in negligible quantities or are not detected, suggesting that their utilization or migration may be more restricted or less stable.

**Figure 2:**
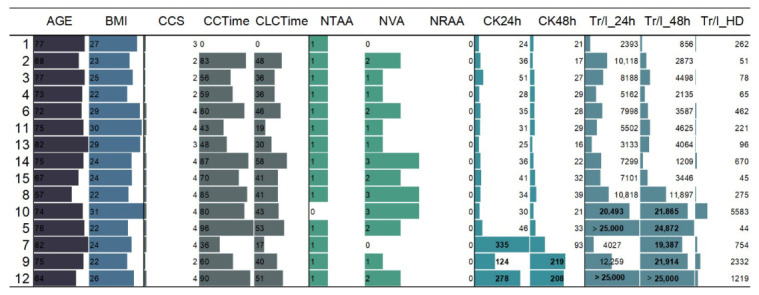
Overview of selected clinical parameters from 15 patients diagnosed with coronary artery disease and treated with coronary artery bypass surgery. **Abbreviations**: CCTime/circulation time (min); CLCTime/clamp cap time (min); NTAA/no of thoracic artery anastomoses; NVA/no of venous anastomoses; NRAA/no of radial artery anastomoses; CK24h/creatinine kinase (U/L), 24 h after the surgency; CK48h/creatinine kinase (U/L), 48 h after the surgency; Tr/I_24 h/Troponin I (ng/L), 24 h after the surgency; Tr/I_48 h/Troponin I (ng/L), 48 h after the surgency; Tr/I_HD/Troponin I (ng/L), Hospital discharge data. **Surgency operation data**: CCS, CLCTime, NTAA, NVA, NRAA; First 24 h (to 24 h after the surgency): CK24h; Tr/I_24h; Second 24 h (to 48 h after the surgency): CK48h; Tr/I_48h; Hospital discharge data: Tr/I_HD.

The analysis of Troponin I level in the first 24 h (to 24 h after surgery) reveals that Patients 5, 7, 9, and 12 demonstrate values exceeding 25,000 (Figure 2). Such high levels strongly indicate significant myocardial injury, likely consistent with acute myocardial infarction. The validity of these findings is reinforced by the observation of high creatine kinase (CK) levels, a finding that is particularly pronounced in Patient 7, who exhibited a CK24h value of 335. The concurrence of Troponin and CK biomarkers serves to substantiate the probability of acute cardiac muscle damage in these subjects. In the majority of cases (10 out of 15), Troponin I levels decreased by the 48 h mark. This tendency was particularly pronounced in patients presenting with early-stage symptoms (Patients 1–6), aligning with the anticipated biological clearance of Troponin I following an acute myocardial event. Typically, Troponin I levels increase within the first 12–24 h following cardiac injury, subsequently declining as the acute phase resolves [13]. However, in 5 out of 15 patients (specifically Patients 7, 8, 9, 10, and 13), Troponin I levels increased at 48 h. Furthermore, these patients exhibited elevated or rising creatine kinase (CK) values, indicating potential ongoing myocardial injury or delayed troponin peak [14]. In another train of thought, the hypothesis that the elderly are more prone to myocardial infarction is one that has been disproven. While patients 1, 3 and 13 do not demonstrate the highest biomarker levels. This finding suggests that age may not be a significant predictor of the severity of heart damage, at least in this particular group. The correlation between body mass index (BMI) and troponin levels is a complex phenomenon that requires further investigation. For instance, Patient 10 has the highest BMI (31) yet only moderate troponin elevations, while Patients 5 and 9, with lower BMIs of 21–22, show significantly elevated cardiac markers. These findings suggest that BMI may not be a reliable standalone predictor of cardiac injury in this context. With regard to the categorization of variables such as NTAA, NVA, and NRAA, the majority of patients exhibit an NTAA value of 1. A higher NVA count (≥2), as observed in Patients 5, 8, 9, 10, and 12, frequently corresponds with elevated troponin and CK levels, suggesting the possibility of underlying vascular complications.

## 3. Discussion

**Figure 3:**
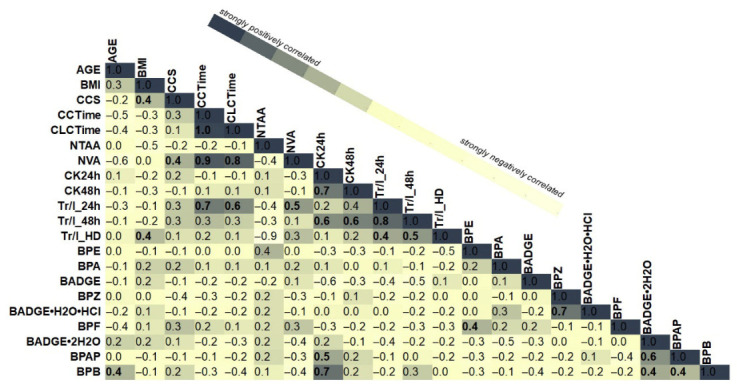
A heat map of the correlation matrix representing associations between clinical variables in 15 coronary artery disease patients and quantified bisphenol levels. CCS/central cord syndrome; CCTime/circulation time (min); CLCTime/clamp cap time (min); NTAA/no of thoracic artery anastomoses; NVA/no of venous anastomoses; NRAA/no of radial artery anastomoses; CK24h/creatinine kinase (U/L), 24 h after the surgency; CK48h/creatinine kinase (U/L), 48 h after the surgency; Tr/I_24h/Troponin I (ng/L), 24 h after the surgency; Tr/I_48h/Troponin I (ng/L), 48 h after the surgency; Tr/I_HD/Troponin I (ng/L), Hospital discharge data.

The heatmap illustrated in Figure 3 displays Pearson correlation coefficients (r) between variables spanning demographic characteristics (AGE, BMI), neurovascular assessments (CCS, CCTime, CLCTime, NTAA, NVA), biochemical markers (CK24h, CK48h, Trp/I_24h, Trp/I_48h, Trp/I_HD), and bisphenol compounds (BPE, BPA, BADGE, BPZ, BADGE·H_2_O·HCl, BPF, BADGE·2H_2_O, BPAP, BPB). Correlation values range from −1 (strong negative correlation) to +1 (strong positive correlation). Darker shading indicates stronger correlations. Values along the diagonal represent perfect self-correlations (r = 1). The implementation of correlation analysis revealed several moderate and strong associations among clinical variables and the levels of bisphenols in the investigated patients. A highly significant positive correlation was identified between the number of venous anastomoses (NVA) and CCTime/circulation time (r = 0.9). In a similar manner, NVA demonstrated a strong correlation with the CLCTime/clamp cap time (min) (r = 0.9). CCTime exhibited a strong positive correlation with Troponin I levels at 24 h (Tr/I_24h) (r = 0.7), while Tr/I_24h also correlated strongly with Tr/I_48h (r = 0.7). Furthermore, a significant positive correlation (r = 0.7) was identified between CK24h and both bisphenol BPB and CK48h. A notable finding was the strong correlation observed between BADGE·H_2_O·HCl and BPZ (r = 0.7). A moderate positive correlation was observed between bisphenol BPB and AGE (r = 0.4). In a similar manner, moderate positive correlations were identified between troponin I levels at hospital discharge (Tr/I_HD) and body mass index (BMI) (r = 0.4), as well as troponin I levels measured at 24 h (TrI/_24h) (r = 0.4) and 48 h (TrI/_48h) (r = 0.5) post-surgery. Troponin I at 24 h (Tr/I_24h) also demonstrated moderate correlations with clamp cap time (CLCTime) (r = 0.6), while TrI/_48h exhibited moderate correlations with both CK24h and CK48h (r = 0.6). A moderate correlation was observed between the number of venous anastomoses (NVA) and central cord syndrome (CCS) (r = 0.4); moreover, a similar correlation was found between BMI and CCS (r = 0.4). With regard to bisphenol compounds, BPE demonstrated moderate correlations with both the number of radial artery anastomoses (NRAA) (r = 0.4) and BPF (r = 0.4). Additionally, the BADGE·2H_2_O demonstrated moderate correlations with both the BPAP and BPB (r = 0.6), whereas the BPAP and BPB exhibited a similar degree of correlation (r = 0.6).

The present study identified several significant correlations between clinical variables, cardiac biomarkers and bisphenol compound levels in patients diagnosed with coronary artery disease. A strong correlation was observed between the procedural parameters of venous anastomosis counts and surgical time, and the incidence of early and late markers of myocardial injury. Furthermore, moderate correlations have been identified between bisphenol exposure and demographic and clinical factors, including age, BMI, and biochemical markers. These findings suggest that environmental exposures, particularly bisphenols, may interact with patient-specific physiological and surgical factors, potentially influencing cardiac outcomes. The results of the study highlight the importance of integrated biomarker and exposure profiling in cardiovascular risk assessment [11].

**Figure 4:**
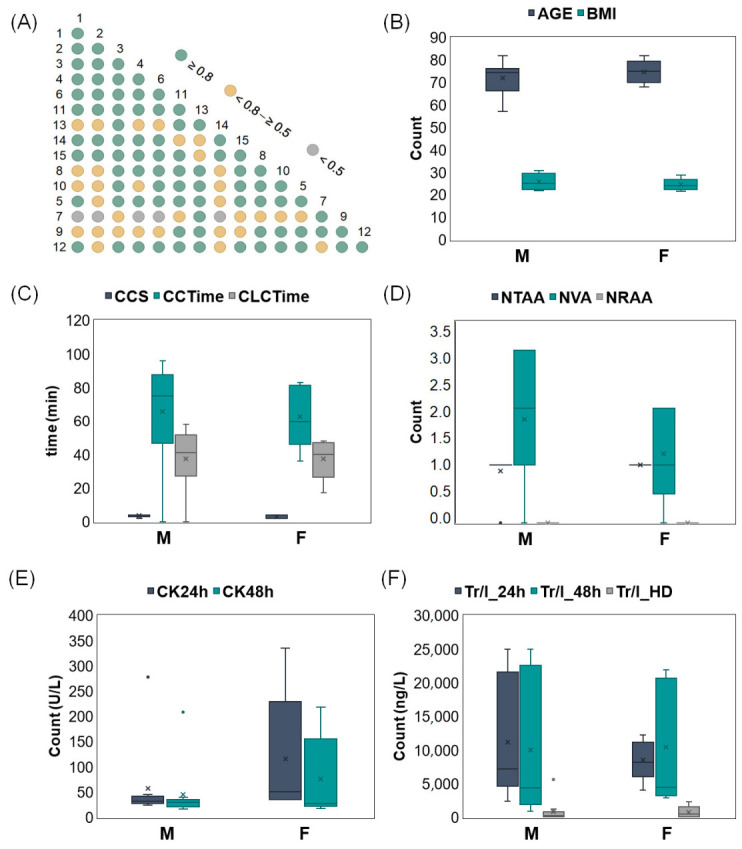
Comparison of clinical parameters and quantified bisphenol levels in a group of 15 patients diagnosed with coronary artery disease, distinguishing between male and female patients. (**A**) Correlation matrix of 15 patients diagnosed with coronary artery disease. Color represent the strength of the Pearson correlation coefficient: green (≥0.8), yellow (0.5–0.8), and gray (<0.5); Boxplots distributions between male and female participants and following paramaters: (**B**) age (AGE) and body mass index (BMI); (**C**) time of central cord syndrome (CCS), circulation (CCTime) and clamp cap time (CLCTime); (**D**) number of thoracic artery anastomoses (NTAA), venous anastomoses (NVA), and radial artery anastomoses (NRAA); (**E**) serum creatine kinase levels (U/L) at 24 h (CK24h) and 48 h post-procedure (CK48h); (**F**) cardiac troponin I concentrations (ng/L) at 24 h (Tr/I_24h), 48 h (Tr/I_48h), and under high-dose conditions (Tr/I_HD).

A correlation matrix was constructed to evaluate the interrelationships among 15 patients diagnosed with coronary artery disease (Figure 4A). Strong positive correlations (r ≥ 0.8) were observed among patients 1, 2, 3, 4, 6, and 11, indicating a high degree of similarity in their clinical and/or biochemical profiles. Moderate correlations (r = 0.5–0.8) were identified among 8, 9, 10, 13 patients, while weaker associations (r < 0.5) suggested more distinct or heterogeneous patterns (patient number 7). These results indicate the existence of groups of patients who may share pathological characteristics [15]. Boxplot analysis of age and body mass index (BMI) (Figure 4B) revealed a similar distribution of age between male and female groups, indicating demographic comparability. However, BMI exhibited a slight increase in males compared with females, which may be associated with differences in clinical parameters or bisphenol exposure profiles. A substantial discrepancy was observed between the two groups with respect to procedural and clinical timing parameters (Figure 4C). A comparison of the results obtained for the males and females revealed that the males exhibited longer and more variable value CCTime (circulation time) and CLCTime (clamp cap time) than the females. In contrast, the values for CCS (central cord syndrome) were low and relatively consistent within both groups. These differences may be attributable to physiological responses that vary with gender [16]. As illustrated in Figure 4D, the distribution of thoracic artery anastomoses (NTAA), venous anastomoses (NVA), and radial artery anastomoses (NRAA) is demonstrated. Patients of the male gender exhibited higher NVA counts and greater interindividual variability compared to patients of the female gender, whereas NTAA and NRAA values remained low and comparable across both groups. Creatinine kinase levels 24 h and 48 h after the emergency (Figure 4E) exhibited distinct profiles between the groups. A significant increase was observed in the female subjects at the 48 h time point, characterized by elevated median values and increased variability in their CK levels when compared to the male subjects, who exhibited comparatively stable CK levels. This delayed elevation in females may suggest prolonged or more intense muscle tissue response or damage, possibly influenced by hormonal or metabolic differences [17]. The temporal distribution of troponin I (TrI) concentrations at 24 h, 48 h, and hospital discharge is shown in Figure 4F. Among male patients, TrI levels were already elevated at 24 h and remained high at 48 h, followed by a slight decrease at the time of discharge. In contrast, female patients demonstrated a delayed rise in TrI, with peak concentrations occurring at 48 h. These results suggest the possibility of gender-specific variations in troponin release dynamics [18].

This study emphasizes substantial discrepancies between males and females with respect to the clinical and biochemical profiles exhibited by patients diagnosed with coronary artery disease [19]. While male subjects demonstrated higher BMI, longer procedural times, and earlier troponin release, female subjects exhibited later but more pronounced increases in CK and TrI, suggesting differences in kinetics and physiological response [20]. The identification of patient group with strong intercorrelations suggests that subgroups may share pathophysiological characteristics, which could inform personalized treatment strategies. The findings emphasize the necessity of considering gender as a physiological variable in cardiovascular diagnostics and management. Further investigation is required in more extensive, diverse patient populations.

## 4. Materials and Methods

### 4.1. Chemical Reagents: Bisphenol Standards and Solvents

All bisphenol standards: CAS: 80-05-7 (bisphenol A), CAS: 620-92-8 (bisphenol F), 2081-08-5 (bisphenol E), 2167-51-3 (bisphenol P), CAS 1675-54-3 (bisphenol A diglycidyl ether; BADGE), CAS: 5581-32-8 (BADGE·2H_2_O), CAS: 4809-35-2 (BADGE·2HCl) with purity ≥ 98.5% were purchased from Sigma Aldrich (Bellefonte, MO, USA).

Solvents used during the experiments and procedure, such as methanol (MeOH), acetonitrile (ACN), dichloromethane (CH_2_Cl_2_) and acetone with LC–MS purity were obtained from Sigma Aldrich (St. Louis, MO, USA). Formic acid (HCOOH) used during HPLC–FLD studies was also obtained from Sigma Aldrich (St. Louis, MO, USA). Deionized water for the HPLC–FLD experiments was obtained in our laboratory using the Hydrolab System, (Gdańsk, Poland). For the LC–MS/MS analysis, water with LC–MS purity was obtained from Merck (Darmstadt, Germany).

The stock solutions of each analyte (5000 ng/mL) were prepared in MeOH in a glass flask and stored in the freezer (−23 °C). The appropriate concentrations were achieved by diluting the primary solution with MeOH.

### 4.2. Sample Collection and Storage

After sternotomy, the first procedure during coronary artery bypass surgery is to collect a sample of fluid from the pericardial sac. Samples were stored in a freezer (−23 °C) and thawed before use in the procedure.

### 4.3. Sample Preparation Procedure

Sample preparation was based on the dispersive liquid–liquid microextraction (DLLME) technique. This procedure was based on previously published work [21].

#### Extraction Recovery Studies, Accuracy, and Precision

Mean recoveries were evaluated at three different concentration levels: 10 ng/mL, 20 ng/mL, and 30 ng/mL of the sample. Mean recovery values were obtained from six replicates for every spiking level. This procedure was described in previously published work [21].

### 4.4. HPLC–FLD: Instrumental Analysis and Chromatographic Conditions

The chromatographic equipment was the same and the gradient settings were set based on the previously published method [21]. The chromatographic equipment consisted of a quaternary pump (Agilent 1200), an autosampler with a thermostat (Agilent 1260 Infinity II Vialsampler), a column thermostat (Agilent 1200), and a fluorescence detector (Agilent 1260). Separation was performed on a Scherzo SM-C18 (150 mm × 4.6 mm) column with a 3 μm particle size (Agilent Technologies, Wilmington, DE, USA), and thermostated at 22 °C. The mobile phase consisted of 50 mM formic acid (HCOOH) in water (component A) and 50 mM HCOOH in acetonitrile (component B). The gradient elution: 0–15 min, component B was increased from 40% to 75%; from 15 to 15.5 min., component B was increased from 75% to 85%; and from 15.5 to 20 min., an isocratic elution with 85% component B. The flow rate was 0.45 mL/min. The samples were thermostated in the autosampler at 8 °C.

The column was washed with 100% component B at a flow rate of 1.0 mL/min after each sample injection, followed by column conditioning with the initial isocratic elution composition for 15 min.

During all chromatographic experiments with application of fluorescence detector a reinforcement 14 or 15 of studied bisphenols was used from 6.5 min to 20 min. Analysis was performed at 4 different excitation wavelengths: 225 nm, 230 nm, 235 nm and 240 nm, with the emission wavelength set at 300 nm. In the HPLC-FLD experiments, the excitation wavelength at 240 nm (or 280 nm) and emission wavelength at 300 nm with signal amplification set at 14 or 15 was found as the most optimal variation taking into account signal strength of analytes at the noise level.

### 4.5. LC–MS/MS Analysis

LC–MS/MS analysis was performed using a triple quadrupole mass spectrometer (8050 Shimadzu, Kyoto, Japan) equipped with LabSolutions version 5.8 software for data collection and instrumental control. Electrospray ionization (ESI−/ESI+) was applied inboth negative and positive ion modes. The ESI–MS/MS spectrometer was coupled with an UHPLC system (LC-30AD binary solvent delivery system, SIL-30AC autosampler, and CTO-20AC thermostat) (Kyoto, Japan). Prior to analysis, the mass spectrometer was calibrated using the manufacturer’s calibration solution. Data were acquired in full scan profile mode (*m*/*z* 50–600). In positive mode, [M+NH_4_]^+^ was selected as the precursor ion, whereas in negative mode, the precursor ion was [M–H]^−^. All BADGEs tend to form adducts in positive ESI mode; namely, ammonium adduct ions were obtained. In negative ESI mode, MeOH–water with no additives was used as the mobile phase to prevent ion suppression.

The ions were detected using multiple reaction monitoring (MRM) mode. Positive and negative ion modes were both employed for compound analysis. The optimization of different MS parameters on the selectivity and MS response (MRM peak areas) for the studied compounds was carried out without a chromatographic column (Supplementary Information Figures S1 and S2, Table S1) [21].

To select the MS/MS parameters, standard solutions of the studied bisphenols at a concentration of 500 ng/mL were infused into the mass spectrometer using a Harvard syringe pump at a flow rate of 10 μL/min. The optimal parameters were as follows: interface temperature 300 °C, DL temperature 250 °C, heat block temperature 400 °C, nebulizing gas flow 2 L/min, drying gas flow 10 L/min, heating gas flow 10 L/min, interface voltage 3.5–4.5 kV, interface current 0.7 μA, and drying gas temperature 290–350 °C. Nitrogen was used as the collision gas, and the energy was set at 20–35 eV. Drying gas flow (N_2_) is crucial for effective ionization and detection. It helps remove solvent vapor from the samples, preventing it from interfering with the ionization process and ensuring accurate measurements. Optimizing drying gas flow in mass spectrometry involves finding a balance between maximizing the analyte signal and maintaining a good peak shape. Such a specific range (290–350 °C) was used to find a balance between efficient desolvation and maintaining the analyte stability. Heating gas flow is a heated nitrogen flow, that facilitates rapid desolvation of droplets to enhance ion evaporation and increase signal intensity. It works with the nebulizer gas to improve sensitivity and prevent source contamination, requiring optimization based on flow rate and solvent composition. 

The chromatographic analysis was performed using a Kinetex C18 analytical column (Phenomenex, Torrance, California, United State) (100 mm × 2.1 mm, 1.7 μm) with column temperature set at 30 °C. The mobile phase consisted of water (mobile phase A) and methanol (mobile phase B) (for BPS, BPF, BPE, BPA, BPAF, BPP, BPZ, BPB, BPAP) and 40 mM ammonium formate in water and methanol (for BADGE·2H_2_O, BADGE·H_2_O, BADGE·H_2_O·HCl, BADGE·2HCl, BADGE). The gradient program for the mobile phase was as follows: 0–1 min, 25% B; 1–5 min, linear to 95% B; then, the system returned to the initial conditions at a flow rate of 0.4 mL/min, with a stop time of 7 min and a post-time of 3 min. The injection volume was set to 1 μL, and the autosampler’s temperature was set at 4 °C.

#### 4.5.1. LC–MS/MS: Method Validation

Validation parameters, including calibration data (calibration equations, linearity presented as a correlation coefficient (R^2^) of the calibration curves, limits of detection (LOD), limits of quantification (LOQ), and precision (RSD) was described in previously published paper [11]. The calibration range for the analyzed bisphenols was 0.04–100 ng/mL, meeting the requirements for the analytical method validation and ensuring accurate determination of linearity within this range. The LOD and LOQ values were calculated using the equations 3.3× (SD/S) and 10× (SD/S), respectively, where SD is the standard deviation of the response (peak area) and S is the slope of the calibration curve (Supplementary Information Table S2).

#### 4.5.2. Fragmentation Pathway of Bisphenols Described by Other Authors

Few studies on the mass spectrometric fragmentation pathway of bisphenols have been reported using high-resolution mass spectrometry (HRMS). Nowadays, by application of modern equipment, e.g., the Orbitrap MS, it is possible to combine the structural formula and secondary mass spectrometry of bisphenols to indicate their likely fracture mode and fracture location [22]. The nine bisphenols gave similar fragmentation patterns and common characteristic neutral losses. The MS/MS fragmentations of nine bisphenols such as BPA, BPB, BPC, BPP, BPF, BPS, BPZ, BPAF, and BPAP, together with several corresponding isotope-labeled compounds, were studied by Orbitrap MS using electrospray ionization (ESI) in negative ion mode and higher energy collisional dissociation (HCD) [22]. It should be noted that the fragmentation pathway of bisphenols has been described by other authors.

### 4.6. Statistical Analysis

All statistical analyses were conducted using Microsoft Excel 2010. To evaluate dissimilarities in bisphenol concentrations measured in pericardial fluid samples from 15 patients, heat map visualizations were created. To further explore underlying relationships within the dataset, a correlation matrix was constructed based on zero-order correlations. Pearson correlation coefficients (r) were calculated to assess linear associations between bisphenol concentrations and related variables, with values interpreted within the conventional range of −1 to +1. A two-tailed *p*-value < 0.05 was considered statistically significant. Box plot analysis was performed to visualize the distribution and variability of bisphenol levels across experimental groups, with a particular focus on gender-dependent differences. Each box plot displayed the median, providing a comparative overview of gender-specific variation in exposure levels.

## 5. Conclusions

The proposed method combines the advantages of DLLME as an extraction technique with HPLC–FLD and LC–ESI–QqQ for the identification and quantification of analyzes in pericardial fluid collected from patients with coronary artery disease undergoing coronary artery bypass surgery. The presented DLLME-based sample preparation technique, coupled with HPLC–FLD and LC–QqQ analysis, provides a reliable analysis of bisphenols in human pericardial fluid.

Strong positive correlations (r ≥ 0.8) were observed among six patients (1, 2, 3, 4, 6, and 11), indicating a high degree of similarity in their clinical and/or biochemical profiles. These results indicate the existence of groups of patients who may share pathological characteristics.

A substantial discrepancy was observed between the two groups with respect to procedural and clinical timing parameters. A comparison of the results obtained for the males and females revealed that the males exhibited longer and more variable values of CCTime (circulation time) and CLCTime (clamp cap time) than the females. In contrast, the values for CCS (central cord syndrome) were low and relatively consistent within both groups. These differences may be attributable to physiological responses that vary with gender.

Patients of the male gender exhibited higher venous anastomoses (NVA) counts and greater interindividual variability compared to patients of the female gender, whereas thoracic artery anastomoses (NTAA) and radial artery anastomoses (NRAA) values remained low and comparable across both groups.

Creatinine kinase levels 24 h and 48 h after the emergency exhibited distinct profiles between the groups. A significant increase was observed in the female subjects at the 48 h time point, characterized by elevated median values and increased variability in their CK levels when compared to the male subjects, who exhibited comparatively stable CK levels. This delayed elevation in females may suggest prolonged or more intense muscle tissue response or damage, possibly influenced by hormonal or metabolic differences. Among male patients, TrI levels were already elevated at 24 h and remained high at 48 h, followed by a slight decrease at the time of discharge. In contrast, female patients demonstrated a delayed rise in TrI, with peak concentrations occurring at 48 h. These results suggest the possibility of gender-specific variations in troponin release dynamics.

It can be anticipated that the results published in the previous [11] and current papers, as well as the results of subsequent experiments and statistical analyses, will enable the finding of connections between the concentrations and types of bisphenols present in pericardial fluid and heart and circulatory system diseases.

## Data Availability

The original contributions presented in this study are included in the article/Supplementary Material. Further inquiries can be directed to the corresponding author.

## Supplementary Materials

The following supporting information can be downloaded at: https://www.mdpi.com/article/10.3390/molecules31091442/s1. Figure S1: QqQ-ESI-MS (top) and MS/MS (bottom) spectra of following bisphenols residues detected in pericardial fluid samples: BADGE (*m*/*z* = 358), BADGE·2HCl (*m*/*z* = 430), BADGE·H_2_O·HCl (*m*/*z* = 412), BADGE·H_2_O (*m*/*z* = 376), BADGE·2H_2_O (*m*/*z* = 394), BPAP (*m*/*z* = 335), BPAF (*m*/*z* = 335), BPZ (*m*/*z* = 267), BPP (*m*/*z* = 345), BPB (*m*/*z* = 241), BPA (*m*/*z* = 227), BPE (*m*/*z* = 213), BPS (*m*/*z* = 249), and BPF (*m*/*z* = 199). Mass spectrometry of studied BPSs. Table S1. MS/MS conditions used for determination and identification of selected bisphenols in biological samples. Figure S2. Representative MRM chromatograms of selected studied bisphenols. BPS (*m*/*z* = 249), BPF (*m*/*z* = 199), BPE (*m*/*z* = 213), BPA (*m*/*z* = 227), BPAF (*m*/*z* = 335), BADGE·2H_2_O (*m*/*z* = 394), BADGE·H_2_O (*m*/*z* = 376), BADGE·H_2_O·HCl (*m*/*z* = 412), BADGE·2HCl (*m*/*z* = 430), BADGE (*m*/*z* = 358). Method Validation. Table S2. Calibration data of detected components including: calibration equations, linearity coefficient (R^2^), LOD, LOQ, and precision (RSD). Fragmentation pathway of bisphenols described by other authors.
