# Supplementary material for: Potential Clinical Application of Determination of Bisphenols in Pericardial Fluid from Patients with Coronary Artery Disease
Source: Molecules. 2026 Apr 27;31(9):1442. doi: 10.3390/molecules31091442 (PMC13165298; doi:10.3390/molecules31091442)
Supplement: Supplementary file 1 [file molecules-31-01442-s001.zip › molecules-4165016-supplementary.pdf]

Article

Potential Clinical Application of Determination of Bisphenols in Pericardial Fluid from Patients with Coronary Artery Disease

Tomasz Tuzimski [1,*], Kamil Baczewski [2], Viorica Railean [3,4], Daria Tarkowska [4,5] and Małgorzata Szultka-Młyńska [4,5]

[1] Department of Physical Chemistry, Faculty of Pharmacy, Medical University of Lublin, Chodźki 4a, 20-093 Lublin, Poland
[2] Department of Cardiac Surgery, Medical University of Lublin, Jaczewskiego 8, 20-093 Lublin, Poland; kamil.baczewski@umlub.edu.pl
[3] Department of Infectious, Invasive Diseases and Veterinary Administration, Institute of Veterinary Medicine, Nicolaus Copernicus University in Torun, Gagarina 7, 87-100 Torun, Poland; viorica.railean@umk.pl
[4] BioColl team, Centre for Modern Interdisciplinary Technologies, Institute of Advanced Studies, Nicolaus Copernicus University, Wilenska 4, 87-100 Torun, Poland; dtarkowska@umk.pl (D.T.); mszultka@umk.pl (M.S.-M.)
[5] Department of Environmental Chemistry and Bioanalytics, Gagarina 7, Faculty of Chemistry, Nicolaus Copernicus University, 87-100 Torun, Poland
* Correspondence: tomasz.tuzimski@umlub.edu.pl; Tel.: +48-814487213

Supplementary Material

Figure S1

QqQ-ESI-MS (top) and MS/MS (bottom) spectra for the following bisphenols residues identified in pericardial fluid samples:

<u>BADGE (*m*/*z* = 358):</u>

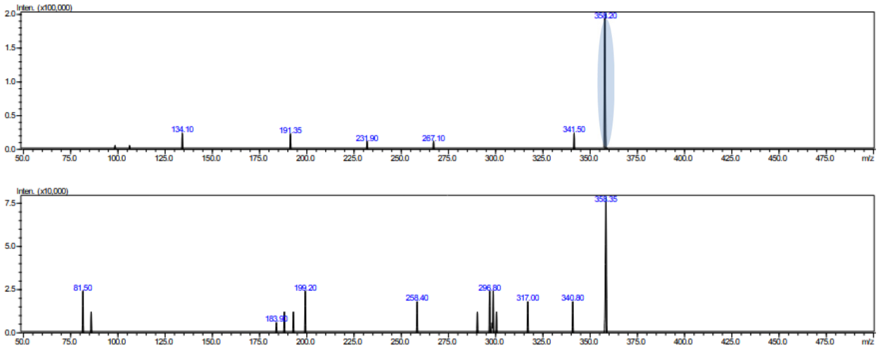

<u>BADGE•2HCl (*m*/*z* = 430):</u>

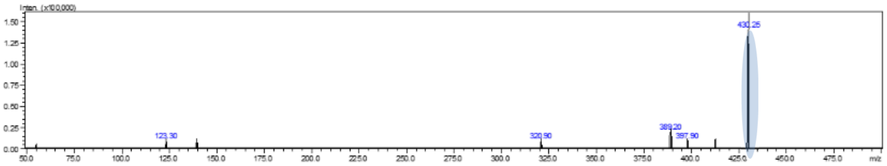

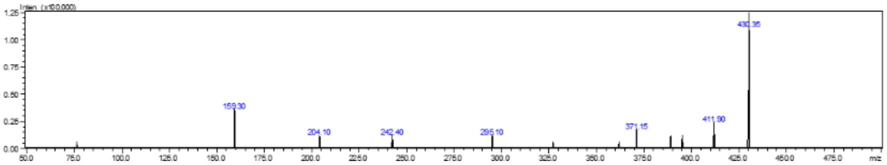

BADGE•H_2O•HCl (*m/z* = 412):

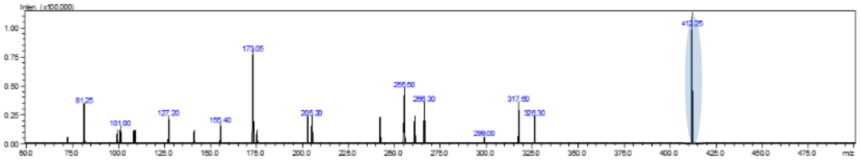

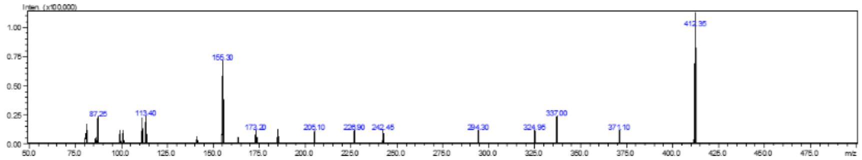

BADGE•H_2O (*m/z* = 376):

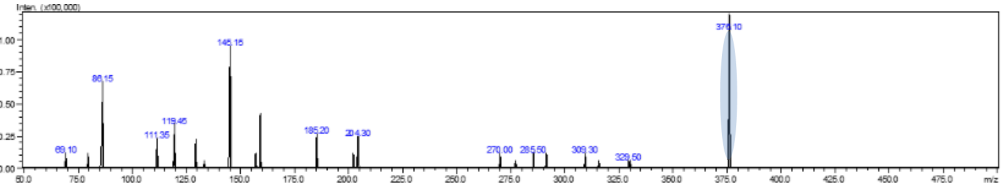

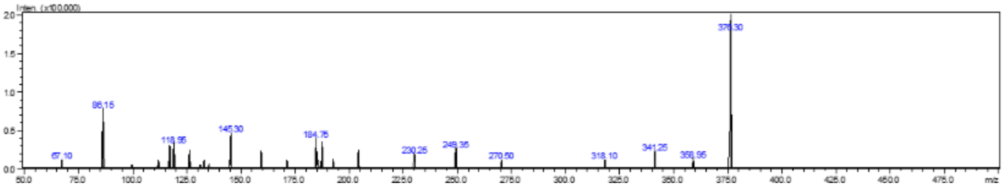

BADGE•$2H_2O$ (*m/z* = 394):

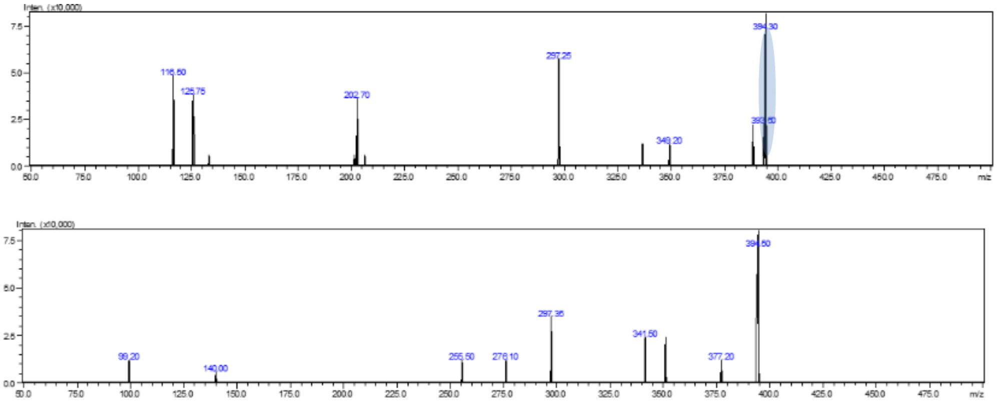

BPAP (m/z = 335):

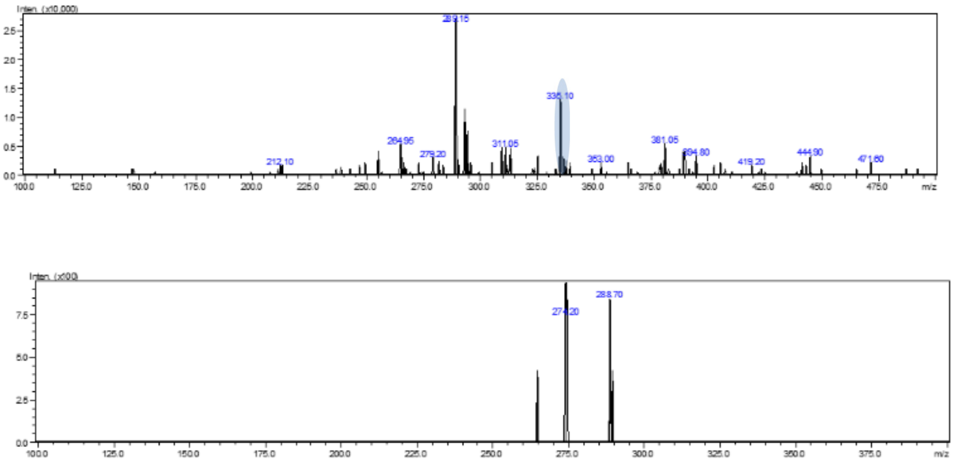

<u>BPAF (m/z = 335):</u>

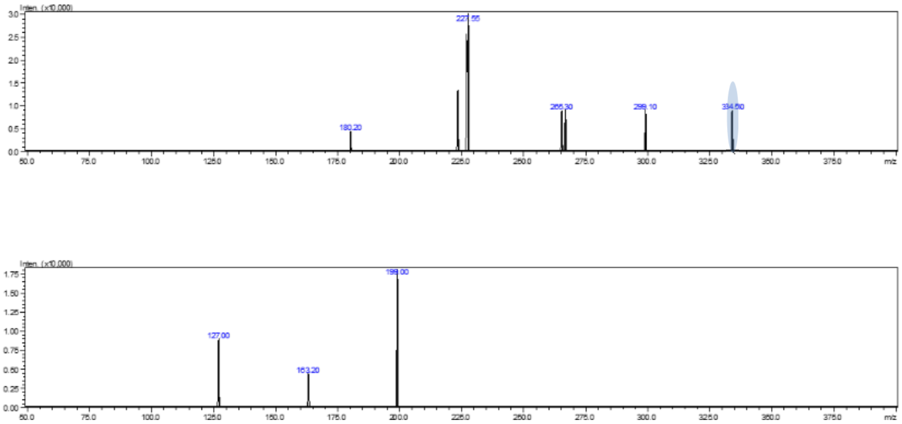

<u>BPZ (m/z = 267):</u>

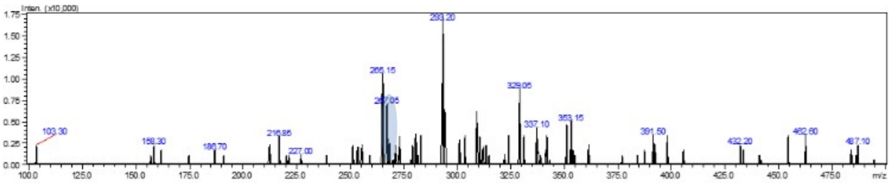

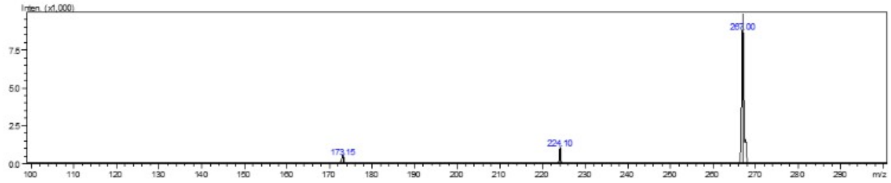

<u>BPP (m/z = 345):</u>

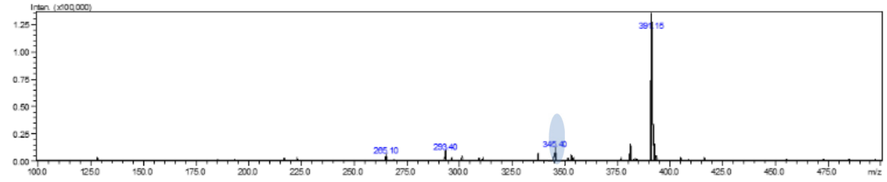

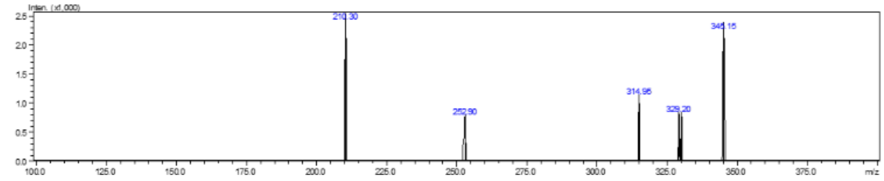

<u>BPB (m/z = 241):</u>

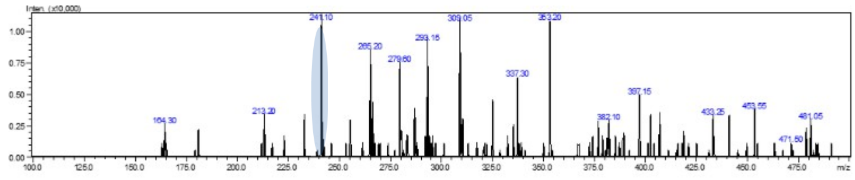

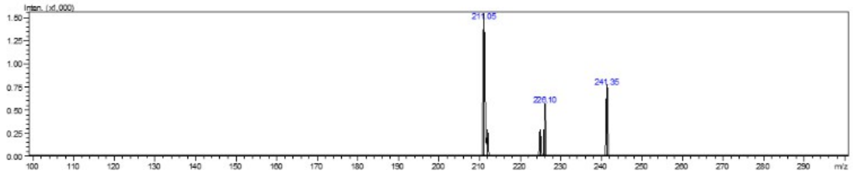

BPA (m/z = 227):

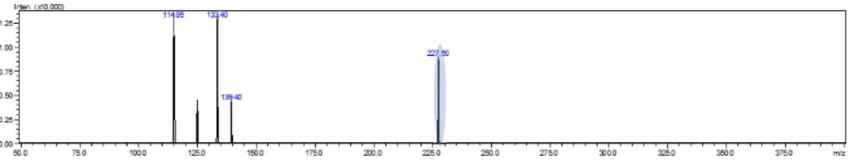

Inten. (x10,000)
114.85
133.40
139.40
227.50
1.25
1.00
0.75
0.50
0.25
0.00
50.0
75.0
100.0
125.0
150.0
175.0
200.0
225.0
250.0
275.0
300.0
325.0
350.0
375.0
m/z

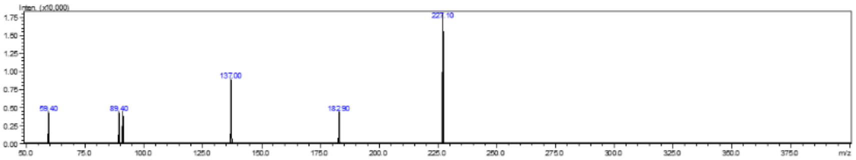

Inten. (x10,000)
227.10
137.00
59.40
89.40
182.90
1.75
1.50
1.25
1.00
0.75
0.50
0.25
0.00
50.0
75.0
100.0
125.0
150.0
175.0
200.0
225.0
250.0
275.0
300.0
325.0
350.0
375.0
m/z

BPE (*m/z* = 213):

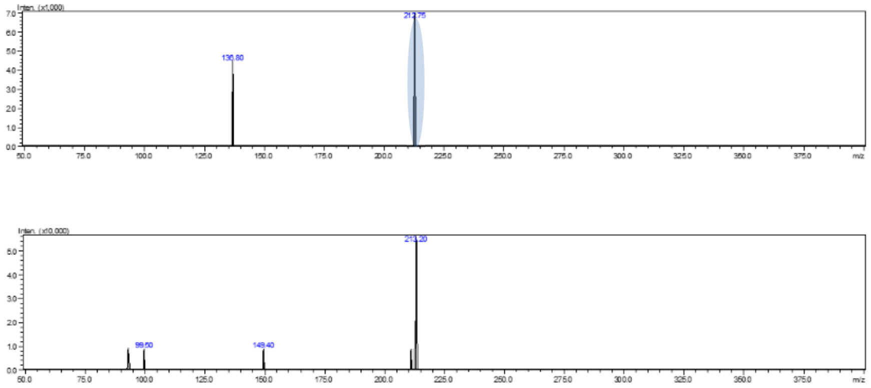

BPS (*m/z* = 249):

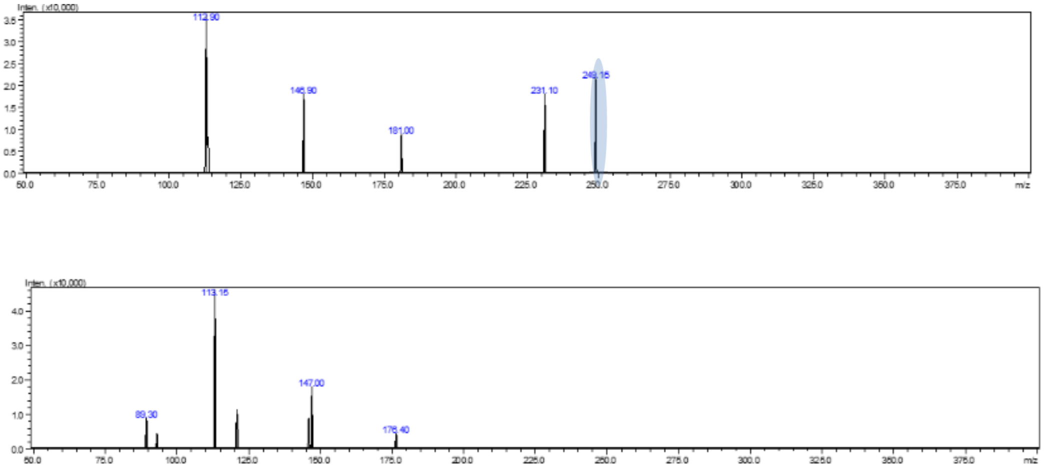

BPF (*m/z* = 199):

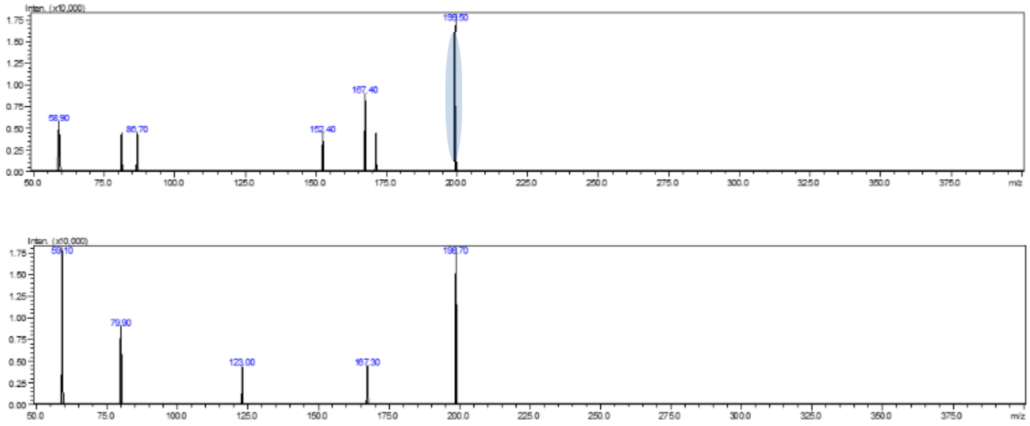

Mass spectrometry of studied BPSs

Table S1 summarizes the two transitions selected for each compound and the optimum collision energies that maximize the intensity of the product ions, used for both quantitative and confirmatory purposes. The obtained data were confirmed from the MS/MS analysis of BPS standards. The MRM mode acquisition was chosen for the quantification of BPSs in this study.

In the case of BADGE and its derivatives (BADGEs), MS/MS spectra of ammonium adducts and the most abundant or most characteristic product ions were recorded. BADGEs showed the characteristic division of the cleavage of the phenyl-alkyl bond with the simultaneous loss of the NH_3. Moreover, two fragment ions characteristic for two different ether chains regarding the hydrolyzed epoxy group were observed. BADGE•H_2O showed an ion at *m/z* 209 corresponding to this fragmentation, and an ion at *m/z* 135, $[C_9H_{11}O]^+$, which contained the epoxy group. Similar fragmentation was obtained for compounds with two hydrolyzed or chloro-hydrozyled epoxy groups (BADGE•$2H_2O$ and BADGE•HCl). A common product ion at *m/z* 135 was observed due to the α-cleavage of the ether group.

BPS contains two phenolic hydroxyl groups with one sulfinyl on each side, and has a higher heat stability than BPA. Hence, in its MS/MS fragmentation spectrum, the ion at *m/z* 156 was generated due to the breakage of two carbon–sulfur bonds and was assigned as $[M\text{-}H\text{-}C_6H_5O]^-$. A product ion at *m/z* 108 was formed by losing one sulfinyl and one phenol. In the MS/MS spectrum of $[M-H]^-$ of BPF, product ions at *m/z* 93 and 105 were assigned to $[M\text{-}H\text{-}C_7H_6O]^-$ and $[M\text{-}H\text{-}C_6H_6O]^-$ respectively. In the MS/MS spectrum of $[M\text{-}H]^-$ of BPA, product ions at *m/z* 211 and 133 were assigned to $[M\text{-}H\text{-}CH_4]^-$ and $[M\text{-}H\text{-}C_6H_6O]^-$, respectively. In the MS/MS spectrum of $[M-H]^-$ of BPAF, product ions at *m/z* 265 and 245 were assigned to $[M\text{-}H\text{-}CHF_3]^-$ and $[M\text{-}H\text{-}CH_6F_3]^-$, respectively.

Table S1. MS/MS conditions used for determination and identification of selected bisphenols in biological samples.

Compound	Parent Ion, *m/z*	Quantifier ion [*Q1*], *m/z* Qualifier ion [Q3], *m/z*	Collision Energy (eV); *Q1, Q3*	Drying Gas Temperature (°C)	Capillary Voltage (V)
**BADGE** *2,2-Bis[4-(glycidyloxy) phenyl]propane Bisphenol A diglycidyl ether*	358	191<br>161	30, 24	320	4500
**BADGE•2HCl** *2,2-Bis [4-(3-chloro-2-hydroxypropoxy)phenyl]propane Bisphenol A Bis(3-chloro-2-hydroxypropyl) ether*	430	227<br>135	26, 29	350	3500
**BADGE•H_2O•HCl** *3-[4-[2-[4-(3-chloro-2-hydroxypropoxy)phenyl]propan- 2-yl]phenoxy]propane-1,2-diol*	412	227<br>191	32, 27	350	4000
**BADGE•H_2O** *3-[4-[2-[4-(oxiran-2-ylmethoxy)phenyl]propan-2-yl]phenoxy]propane-1,2-diol*	376	209<br>135	29, 31	320	3500
**BADGE•$2H_2O$** *2,2-Bis [4-(2,3-dihydroxypropoxy)pheny]propane Bisphenol A Bis(2,3-dihydroxypropyl) ether*	394	209<br>135	25, 29	320	3500
**BPAP** *4-[1-(4-hydroxyphenyl)-1- phenylethyl]phenol*	335	289<br>265	33, 30	290	4500
**BPAF** *4-[1,1,1,3,3,3-hexafluoro-2-(4- hydroxyphenyl)propan-2-yl]phenol*	335	265<br>245	31, 28	290	4500
**BPZ** *4-[1-(4- hydroxyphenyl)cyclohexyl]phenol*	267	173<br>145	26, 33	320	3500
**BPP** *4-[2-[4-[2-(4-hydroxyphenyl)propan-2-yl]phenyl]propan-2-yl]phenol*	345	330<br>131	28, 29	320	4000
**BPB** *4-[2-(4-hydroxyphenyl)butan-2- yl]phenol*	241	226<br>212	29, 30	290	4500
**BPA** *2,2-Bis(4-hydroxyphenyl) propane*	227	211<br>133	29, 30	290	4500

**BPE** *4-[1-(4-hydroxyphenyl)ethyl]phenol*	213	198<br>119	29, 35	320	4000
**BPS** *Bis(4-hydroxyphenyl)sulfone*	249	108<br>156	22, 35	320	3500
**BPF** *4,4 -Methylenediphenol*	199	93<br>105	27, 24	320	4000

After determining the best conditions for isolating the precursor (analyte proton adduct), full scan MS/MS mode was used to record product ions from the standard solution of each target compound. The fragmentation amplitude and isolation width for each analyte were manually optimized to increase the method's selectivity and sensitivity and to select the most intense and characteristic fragmentation ions for qualitative analysis and one of the highest intensities for quantitative analysis. Exemplarily, MRM chromatograms of the studied bisphenols are presented in **Figure S2**.

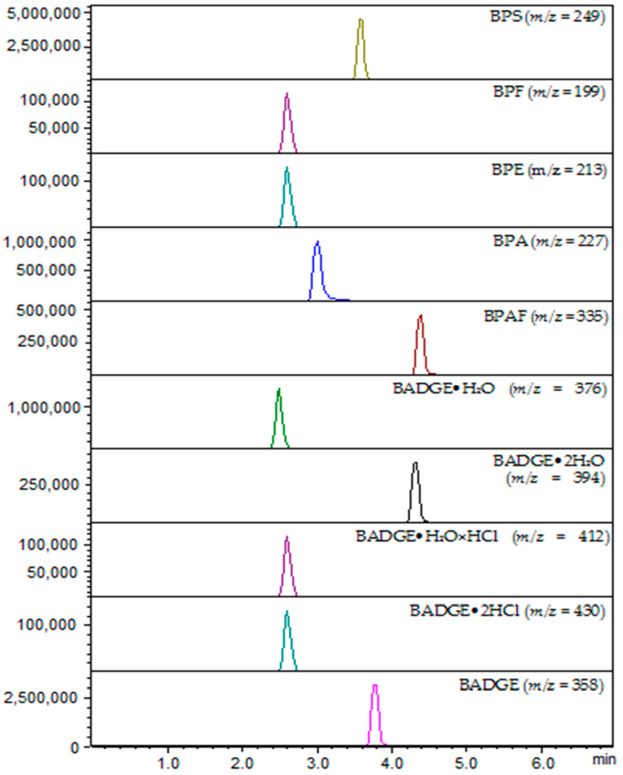

Figure S2. Representative MRM chromatograms of selected studied bisphenols. BPS (*m/z* = 249), BPF (m/z = 199), BPE (*m/z* = 213), BPA (*m/z* = 227), BPAF (*m/z* = 335), BADGE•2H_2O (*m/z* = 394), BADGE•H_2O (*m/z* = 376), BADGE•H_2O•HCl (*m/z* = 412), BADGE•2HCl (*m/z* = 430) and BADGE (*m/z* = 358).

Method Validation

The method was validated according to ICH guidelines. Validation parameters, such as calibration data, including calibration equations, linearity presented as a correlation coefficient (R^2) of the calibration curves, limits of detection (LOD) and quantification (LOQ), and precision (RSD), are presented in **Table S2**.

Table S2. Calibration data of detected components, including calibration equations, linearity coefficient (R^2), LOD, LOQ, and precision (RSD).

Compound	Regression Equation	R^2	RSD%	LOD (ng/mL)	LOQ (ng/mL)
BADGE	y = 5043 + 308	0.9997	2.52	0.11	0.33
BADGE•2HCl	y = 3816 + 869	0.9998	1.86	0.15	0.45
BADGE•H_2O•HCl	y = 4787 + 289	0.9993	1.46	0.21	0.63
BADGE•H_2O	y = 11,663 + 149	0.9996	0.93	0.28	0.84
BADGE•$2H_2O$	y = 5025 + 511	0.9996	1.11	0.37	1.11
BPAP	y = 6150 + 115	0.9997	1.96	0.09	0.27
BPAF	y = 4730 + 435	0.9990	1.90	0.06	0.18
BPZ	y = 5910 + 258	0.9993	1.84	0.21	0.63
BPP	y = 4920 + 105	0.9995	0.97	0.03	0.09
BPB	y = 8149 + 507	0.9998	2.12	0.33	0.69
BPA	y = 9721 + 603	0.9993	2.15	0.06	0.18
BPE	y = 7495 + 1740	0.9995	1.03	0.04	0.12
BPS	y = 15,061 + 5799	0.9998	2.42	0.27	0.81
BPF	y = 5993 + 348	0.9995	2.34	0.21	0.63

Fragmentation pathway of bisphenols described by other authors

By application of more specialistic equipment, e.g., the Orbitrap MS, it is currently possible to combine the structural formula and secondary mass spectrometry of bisphenols to indicate their likely fracture mode and fracture location. The nine bisphenols gave similar fragmentation patterns and common characteristic neutral losses. The MS/MS fragmentations of nine bisphenols such as BPA, BPB, BPC, BPP, BPF, BPS, BPZ, BPAF, and BPAP, together with several corresponding isotope-labeled compounds, were studied by Orbitrap MS using electrospray ionization (ESI) in negative ion mode and higher energy collisional dissociation (HCD) [.

Authors have described in detail the elemental compositions of the ions, which were calculated from the accurate mass data. Authors have aslo described in great detail and proposed in figures the fragmentation pathway of $[M–H]^-$ derived from bisphenols in negative ion mode such as BPA, BPF, BPS and BPP, in addition to the fragmentation pathway of $[M–H]^-$ derived from the following bisphenols in negative ion mode: BPF, BPS, BPZ, BPAF, and BPAP.

Common MS/MS product ions and characteristic neutral losses were summarized. Six bisphenols formed the common product ion at m/z 93 (C_6H_5O). The $[M - H]^-$ ions of five bisphenols were found to lose a phenol group (C_6H_5OH). Four bisphenols formed the $[M - H - CH_4]^-$ ion. The proposed fragmentation pathways of representative compounds of BPA and BPS were verified from the analysis of isotope-labeled compounds. The analyses of isotope-labeled compounds were consistent with those of the corresponding unlabeled bisphenols. As the authors rightly summarized, the described results of fragmentations should provide valuable information for the structural characterization of similar compounds and their metabolites in vitro and in vivo.
